# Anteroposterior Diameter Is Associated with Conversion from Right Minithoracotomy to Median Sternotomy in Minimally Invasive Cardiac Surgery

**DOI:** 10.3390/jpm15080353

**Published:** 2025-08-04

**Authors:** Quynh Nguyen, Durr Al-Hakim, Richard C. Cook

**Affiliations:** 1 Division of Cardiac Surgery, Department of Surgery, University of British Columbia, Vancouver, BC V6Z 1Y6, Canada; 2 School of Biomedical Engineering, University of British Columbia, Vancouver, BC V6T 1Z4, Canada; 3 Faculty of Medicine and Dentistry, University of British Columbia, Vancouver, BC V6T 1Z4, Canada; durra@student.ubc.ca; 4 Division of Cardiac Surgery, Vancouver General Hospital, 899 West 12th Avenue, Vancouver, BC V5Z 1M9, Canada

**Keywords:** minimally invasive cardiac surgery, anteroposterior dimension, mitral valve repair, minithoracotomy

## Abstract

**Background**: Minimally invasive cardiac surgery (MICS) via right minithoracotomy is a safe, reproducible approach with excellent outcomes and reduced costs compared to median sternotomy. Despite careful patient selection, conversion to sternotomy occurs in 1–3% of cases and is associated with significantly higher morbidity and mortality. Small body habitus, particularly a short anteroposterior (AP) diameter, may increase the risk of conversion, but this has not been previously studied. This study aims to identify preoperative factors associated with conversion to improve patient selection for MICS. As cardiovascular surgery becomes increasingly personalized, identifying anatomical factors that predict technical complexity is essential. **Methods**: This retrospective study included 254 adult patients who underwent elective MICS between 2015 and 2024 at a tertiary hospital. Patient characteristics, computed tomography (CT) scans, intraoperative parameters, and postoperative outcomes were reviewed. AP diameter was defined as the distance from the posterior sternum to the anterior vertebral body at the mitral valve level on CT. Statistical analyses included Mann−Whitney and Fisher’s exact/chi-square tests. **Results**: Conversion to sternotomy occurred in 1.6% of patients (n = 4). All converted patients were female. The converted group had a significantly shorter median AP diameter (100 mm vs. 124 mm, *p* = 0.020). Conversion was associated with higher rates of stroke and infection (25.0% vs. 0.8%, *p* = 0.047 for both), but no significant differences in hospital stay, bleeding, or renal failure. **Conclusions:** An AP diameter of less than 100 mm was associated with a higher risk of conversion to sternotomy in MICS. Incorporating simple, reproducible preoperative imaging metrics into surgical planning may advance precision-guided cardiac surgery and optimize patient outcomes.

## 1. Introduction

Minimally invasive cardiac surgery (MICS) through a right minithoracotomy has gained widespread adoption as an alternative to traditional median sternotomy, offering a safe and reproducible approach for various cardiac procedures. Studies have shown that MICS provides equivalent short- and long-term outcomes compared to median sternotomy, while also offering several advantages, including decreased use of blood products, lower infection rates, shorter hospital stays, faster postoperative recovery, and reduced healthcare costs [1,2,3,4]. These benefits have contributed to its increasing utilization in appropriately selected patients.

In recent years, the demand for less invasive surgical options has grown as patients and providers seek to reduce surgical trauma, hospital stays, and recovery time without compromising outcomes. MICS has emerged as a key component of this trend, offering reduced perioperative morbidity while maintaining equivalent long-term efficacy. However, the success of MICS is highly dependent on careful patient selection and preoperative planning, particularly in identifying those at risk for intraoperative challenges.

Despite careful preoperative planning and patient selection, conversion to median sternotomy remains necessary in a subset of cases. Large series have reported conversion rates ranging from 1–3%, with the most frequent causes including bleeding, pulmonary adhesions, vascular complications such as type A aortic dissection, and poor surgical exposure [3,5]. While conversion is sometimes unavoidable, it is associated with significantly higher morbidity and mortality [5].

Although several clinical and anatomical factors have been associated with conversion to sternotomy, quantifiable imaging-based predictors remain poorly defined. In particular, thoracic dimensions may directly influence surgical exposure and technical feasibility. As cardiac surgery continues to evolve toward personalized approaches, incorporating objective, reproducible imaging parameters into preoperative assessment may help reduce conversion risk and optimize outcomes. One potential risk factor for conversion is small body habitus, particularly a reduced anteroposterior (AP) diameter. A shorter AP distance may limit surgical exposure and maneuverability, increasing technical difficulty and the likelihood of conversion. However, this association has not been previously studied.

This study aims to identify preoperative factors, including AP diameter, that are associated with conversion to median sternotomy. By refining patient selection criteria, we aim to minimize conversion rates, enhance surgical planning, and improve the overall safety and efficacy of MICS. As cardiovascular surgery moves toward personalized and precision-guided approaches, incorporating patient-specific anatomical characteristics into procedural planning becomes increasingly critical.

## 2. Materials and Methods

The study protocol was reviewed and approved by the Health Research Ethics Board at the University of British Columbia, Vancouver, Canada. The ethics board waived the requirement for individual patient consent due to the retrospective design of the study.

This study included 254 consecutive adult patients who underwent minimally invasive cardiac surgery (MICS) via right minithoracotomy between 2015 and 2024 at a tertiary hospital. All procedures were performed by a single surgeon. MICS included mitral and tricuspid valve repairs and replacements, atrial septal defect (ASD) repairs, patent foramen ovale (PFO) closures, myxoma resections, and combined procedures. During this period, the surgeon performed a total of 534 cases encompassing these procedures; 254 (48%) were performed via minimally invasive right minithoracotomy and 280 (52%) via planned median sternotomy. Patient baseline clinical characteristics, intraoperative parameters, and postoperative outcomes were collected through chart review. Preoperative parameters were based on the most recent available data before the surgery date.

**Figure 1:**
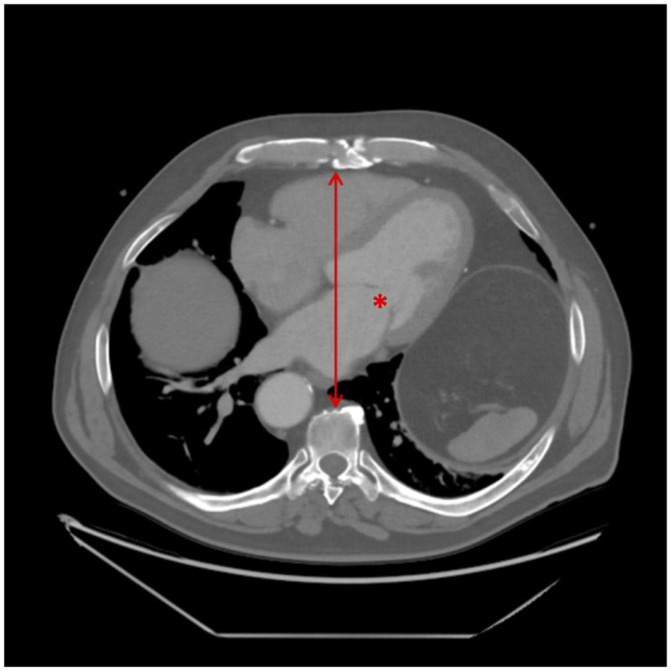
Measurement of anteroposterior (AP) diameter on preoperative CT imaging. The asterisk (*) indicates the mitral valve at the approximate level of A2/P2. The line with arrowheads represents the anteroposterior distance, measured from the posterior aspect of the sternum to the anterior surface of the corresponding vertebral body.

Complex surgical procedures were defined as complex valve repair or combined surgeries, such as double valve procedures. Anteroposterior (AP) diameter/distance was measured from the posterior table of the sternum to the anterior aspect of the vertebral body at the level of the mitral valve on CT scans (Figure 1). Renal failure was defined using the Kidney Disease: Improving Global Outcomes (KDIGO) criteria, as follows: (i) an increase in serum creatinine by ≥26.5 µmol/L within 48 h, (ii) an increase to ≥1.5 times baseline within 7 days, or (iii) urine output <0.5 mL/kg/h for 6 h.

Non-parametric continuous variables were expressed as medians with interquartile ranges (IQRs), while categorical variables were presented as absolute numbers with corresponding percentages. The Mann−Whitney test was used for non-parametric continuous data, and categorical variables were analyzed using Fisher’s exact test or the chi-square test.

A *p*-value of less than 0.05 was considered statistically significant. All analyses were performed using SPSS version 28.0.0.1 (IBM SPSS Statistics, Armonk, New York, NY, USA).

### 2.1. Surgical Technique

All procedures were performed via a 5–6 cm right minithoracotomy, typically in the 3rd or 4th intercostal space. The pericardium was opened and secured with stay sutures to optimize exposure. CO_2_ insufflation was routinely used throughout the procedure to prevent the accumulation of room air in the left side of the heart. A 10 mm “endocameleon” scope (Storz), which provides 0–90-degree angles of visualization, was placed through a separate incision 2 interspaces below the minithoracotomy incision. This was used to aid with visualization; however, most procedures were performed using direct vision through the minithoracotomy incision. When needed, a pledgeted suture was placed onto the fibrous portion of the right hemi-diaphragm, and brought out of the chest through a separate stab incision, to move the diaphragm caudad. A weighted sucker was placed into the left atrium via the right superior pulmonary vein to clear blood from the left atrium. At the end of the procedure, this was replaced with a left ventricular vent, which was placed through the mitral valve.

Femoral arterial and venous cannulation were routinely performed for cardiopulmonary bypass. In the early experience, a cut-down through a 2–3 cm incision was used to expose the anterior surfaces of the femoral artery and vein, and 4–0 polypropylene purse-strings were placed prior to placement of the cannulae using the Seldinger technique. In the more recent experience, percutaneous cannulation using ultrasound guidance became the preferred method of femoral cannulation. Common femoral arterial access was typically on the right side, using the “preclose” technique with 2 ProGlide sutures. Femoral venous access was typically on the left side, as the vein was typically larger on the left with the patient positioned with the right side slightly up for the right minithoracotomy approach. The position of the cannula in the right atrium was confirmed by transesophageal echocardiography (TEE). Venous drainage was augmented with an additional superior vena cava (SVC) cannula placed percutaneously via the right internal jugular vein in patients > 90 kg.

Antegrade and retrograde cold blood cardioplegia were administered for most cases. The retrograde catheter was placed into the coronary sinus using TEE guidance. The initial dose of cardioplegia was an antegrade dose of 1 L of cold blood cardioplegia. This was supplemented with 300–500 cc of cold blood administered retrogradely every 20 min while the heart was cross clamped. A Chitwood clamp was applied through a separate stab incision to the distal ascending aorta under direct vision through the thoracotomy for aortic cross-clamping

### 2.2. Postoperative Care

Postoperatively, all patients were transferred directly to the cardiac surgery intensive care unit (ICU), with a plan for early extubation within 4 h of ICU arrival. Once extubated and hemodynamically stable off vasoactive agents, patients were transferred to the cardiac surgery recovery ward, typically within 24 h. Routine ward care included analgesia, diuresis, reintroduction of cardiac medications, and early mobilization. Patients were discharged once vital signs were within the normal range, they were euvolemic, tolerating oral intake, and had returned to baseline mobility. All patients were referred to a cardiac rehabilitation program upon discharge.

## 3. Results

### 3.1. Conversion Rate from MICS to Median Sternotomy

Four patients (1.6%) required conversion from MICS to median sternotomy, hereafter referred to as the “converted” group, while the remaining 250 patients (98.4%) constituted the “non-converted” group. The reasons for conversion included difficult cannulation in one patient, challenging exposure of the mitral valve anatomy in one patient, and persistent mitral regurgitation (MR) following repair in two patients.

### 3.2. Patient Characteristics

Baseline characteristics were well balanced between the converted and non-converted groups (Table 1). All patients in the converted group were female (100% vs. 32.3%, *p* = 0.019). There were no significant differences in body mass index (BMI), preoperative heart function, or renal function between the two groups. However, the non-converted group had a higher prevalence of severe MR compared to the converted group (82% vs. 25%, *p* = 0.001).

Patients in the converted group had significantly shorter AP diameters. The median AP diameter in the converted group was 100 mm (IQR 93–105 mm), compared to 124 mm (IQR 109–143 mm) in the non-converted group (*p* = 0.020).

### 3.3. Intraoperative Parameters

There were no significant differences in intraoperative parameters between the two groups (Table 2). The proportion of complex surgery was comparable between both groups. The majority of MICS cases involved mitral valve (MV) repair, with 168 patients (67%) in the non-converted group and 2 patients (50%) in the converted group undergoing repair. None of the converted patients required valve replacement instead of a planned valve repair. Skin-to-skin operative time, blood product usage, and pain control strategies were similar between the two groups.

### 3.4. Postoperative Outcomes

Both groups had a median hospital stay of 6 days following surgery (Table 3). Postoperative stroke occurred more frequently in the converted group (n = 1) compared to the non-converted group (n = 2, *p* = 0.047). Similarly, postoperative infection rates were higher in the converted group (n = 1) than in the non-converted group (n = 2, *p* = 0.047). Renal failure occurred in 15 patients in the non-converted group and one patient in the converted group, with only the converted patient requiring renal replacement therapy with intermittent hemodialysis (*p* = 0.230). Valve regurgitation outcomes were comparable between the two groups, and there were no significant differences in lymphocele formation at follow-up.

## 4. Discussion

In our study, the rate of conversion from minimally invasive cardiac surgery (MICS) to median sternotomy was 1.6%, which is consistent with previously reported rates of 1–3% in atrioventricular valve surgery [2,4,5,6,7]. The primary reasons for conversion included persistent MR following attempted repair (n = 2), difficult exposure of the mitral valve anatomy (n = 1), and difficult cannulation (n = 1). Persistent MR may reflect complex valve pathology or difficult exposure, both of which pose significant challenges in a minimally invasive approach. While lateral minithoracotomy typically provides better valve visualization, exposure in our case was significantly limited due to abnormal valve anatomy including leftward valve displacement, a shortened AP dimension, and scarred leaflets suggestive of prior resolved endocarditis, necessitating conversion to sternotomy. Difficult cannulation is another important consideration in MICS, as limited access increases the technical complexity of establishing cardiopulmonary bypass. Patients with tortuous or calcified vessels are particularly at risk, as these anatomical factors can increase the likelihood of vascular complications and necessitate conversion [7].

The low conversion rate in our study reflected a deliberate shift in institutional practice after July 2019, when we began avoiding MICS in patients with an intrathoracic AP diameter of less than 100 mm on preoperative chest CT imaging, or other anatomical risk factors such as severe pectus excavatum. This decision was informed by surgical experience and patterns observed in anatomically complex repairs at our institution. Our findings support the role of precision surgical planning, where preoperative CT imaging enables patient-specific risk stratification, aligning with the broader movement toward personalized medicine in cardiovascular care.

Our findings indicate that an intrathoracic AP diameter of less than 100 mm, measured at the level of the mitral valve, was significantly associated with conversion. A smaller thoracic cavity limits instrument maneuverability and hinders optimal visualization, increasing the technical difficulty of MICS. This constraint may be further exacerbated by additional anatomical factors such as pleural adhesions, severe pectus excavatum, or complex valve pathology, all of which increase the risk of conversion [5,7]. Notably, all converted patients were female, likely due to sex-based anatomical differences, as female patients generally have smaller thoracic cavities and shorter AP diameters [8]. However, the relative infrequency of conversion limits the strength of subgroup analyses and generalizability of this finding.

Our study highlights the important role of preoperative CT imaging in MICS patient selection. Chest CT is already standard practice for preoperative planning and has been used in various minimally invasive cardiac procedures to predict procedural complexity and guide decision-making. Prior studies in robotic minimally invasive direct coronary artery bypass have demonstrated the role of CT in measuring the lateral distance of the left anterior descending artery from the sternum and its depth within pericardial fat, both of which were found to predict conversion risk [9,10]. Similarly, in minimally invasive mitral valve surgery, three-dimensional CT imaging has been used to evaluate cannulation sites and optimize surgical access [11]. Additionally, parameters such as chest width and access angle have been identified as predictors of procedural complexity in other minimally invasive and endoscopic valve surgery studies [12,13]. Our study builds on these applications by demonstrating that intrathoracic AP diameter, measured via CT, is a simple and reproducible metric for identifying patients at increased risk of conversion during MICS. Further advances, including automated CT scan analysis using artificial intelligence, may enable even more refined patient selection and enhance the precision of minimally invasive cardiac surgery.

Converted patients had higher rates of postoperative complications compared to non-converted patients, with a higher incidence of stroke and infection. However, no significant differences were observed in other outcomes, including hospital length of stay (LOS), reoperation for bleeding, renal failure, or vascular complications, as demonstrated by the similar rate of lymphocele formation at follow-up [3,5,7]. These findings are consistent with previous reports in the literature. However, we acknowledge that our small sample size and reduced statistical power limits the ability to draw definitive conclusions about these rare complications in the context of prior studies.

## 5. Limitations

The limitations of this study included those inherent to its single-center retrospective design, which may impact the generalizability of our findings. Given the predominance of mitral valve procedures in our cohort, our findings are most reflective of predictors of procedural complexity and conversion risk in minimally invasive mitral valve surgery. Additionally, the small sample size of converted patients limits the strength of conclusions that can be drawn due to reduced statistical power and constrained subgroup analyses, and may affect the generalizability of our findings. The decision to convert may also be influenced by the surgeon’s experience, including their expertise in minimally invasive techniques, threshold for conversion in the context of limited exposure, and ability to manage major intraoperative complications. Furthermore, institutional factors such as team experience and access to specialized MICS instrumentation can introduce variability. The lack of inter-rater reliability assessment for AP diameter measurements represents an additional methodological limitation. Furthermore, unaccounted factors that were not included in our analysis may have played a role in conversion, warranting further investigation in larger, multi-center studies.

## 6. Conclusions

In this retrospective single-surgeon study, we found that a reduced AP thoracic diameter upon preoperative CT was associated with conversion from MICS to sternotomy. These results support the use of preoperative imaging metrics as a practical and reproducible way to improve patient selection for MICS, particularly in complex mitral valve or combined cases. As imaging becomes more integrated into surgical workflows, objective anatomical predictors such as AP diameter may contribute to safer and more tailored operative planning.

This study is limited by its single-center, retrospective nature and the small number of converted cases, which constrain statistical power and limit generalizability. Additionally, we did not formally assess inter-rater reliability of AP measurements or explore other thoracic dimensions. The threshold of 100 mm was derived empirically from our institutional experience and warrants prospective validation.

Future directions include validating this metric in larger, multi-center cohorts, and evaluating additional anatomical predictors. These tools may help refine risk stratification and promote the broader application of precision-guided cardiac surgery.

## Figures and Tables

**Table 1:** Patient baseline clinical characteristics.

Parameters	Non-Converted (N = 250)	Converted (N = 4)	Total (N = 254)	*p* Value
**Demographics**				
Age	63 (52–72)	55 (40–68)	63 (51–72)	0.579
Female	82 (33)	4 (100)	86 (34)	**0.019**
BMI (kg/m^2^)	25 (22–29)	23 (21–26)	25 (22–29)	0.570
**Co-morbidities**				
NYHA class				0.210
I	73 (29)	0 (0)	73 (29)	
II	101 (40)	1 (25)	102 (40)	
III	60 (24)	2 (50)	62 (24)	
IV	16 (6)	1 (25)	17 (7)	
**Lab and imaging**				
Creatinine (umol/L)	86 (72–99)	71 (70–79)	86 (72–99)	0.250
EF (%)				0.785
>50	223 (89)	4 (100)	227 (89)	
41–49	17 (7)	0 (0)	17 (7)	
<40	10 (4)	0 (0)	10 (4)	
MR				**0.001**
None/trace	10 (4)	1 (25)	11 (4)	
Mild	8 (3)	0 (0)	8 (3)	
Moderate	16 (6)	2 (50)	18 (7)	
Severe	206 (82)	1 (25)	207 (82)	
TR				0.411
None/trace	105 (42)	1 (25)	106 (42)	
Mild	80 (32)	1 (25)	81 (32)	
Moderate	47 (19)	1 (25)	48 (19)	
Severe	14 (6)	1 (25)	15 (6)	
AP dimension (mm)	124 (109–143)	100 (93–105)	124 (108–143)	**0.020**

AP, anterior-posterior; BMI, body mass index; EF, ejection fraction; MR, mitral regurgitation; NYHA, New York Heart Association; TR, tricuspid regurgitation.

**Table 2:** Intraoperative parameters.

Parameters	Non-Converted (N = 250)	Converted (N = 4)	Total (N = 254)	*p* Value
**Urgency**	29 (12)	1 (25)	30 (12)	0.397
**Redo surgery**	21 (8)	1 (25)	22 (9)	0.306
**Complex surgery**	131 (52)	2 (50)	133 (52)	1.000
**Procedures**				0.432
MV repair	168 (67)	2 (50)	170 (67)	
MV replacement	27 (11)	0 (0)	27 (11)	
TV repair	5 (2)	0 (0)	5 (2)	
TV replacement	5 (2)	1 (25)	6 (2)	
ASD repair	16 (6)	1 (25)	17 (7)	
Tumor resection from MV	1 (1)	0 (0)	1 (1)	
**Combined surgery**	27 (11)		27 (11)	1.000
MV repair/TV repair	6 (2)	0 (0)	6 (2)	
MV repair/ASD repair	5 (2)	0 (0)	5 (2)	
MV replacement/TV repair	2 (1)	0 (0)	2 (1)	
MV replacement/ASD repair	3 (1)	0 (0)	3 (1)	
MV repair/PFO closure	7 (3)	0 (0)	7 (3)	
MV replacement/PFO closure	3 (1)	0 (0)	3 (1)	
TV replacement/PFO closure	1 (1)	0 (0)	1 (1)	
**Skin-to-skin time (hour)**	5 (4–5)	5 (3–6)	5 (4–5)	0.681
**Blood product**				
pRBC	42 (17)	2 (50)	44 (17)	0.140
Cryoprecipitate	2 (1)	0 (0)	2 (1)	1.000
Fresh frozen plasma	27 (11)	2 (50)	29 (11)	0.065
Platelet	73 (29)	3 (75)	76 (30)	0.081
**Pain control**				
Paravertebral block	5 (2)	0 (0)	5 (2)	1.000
Paravertebral catheter	174 (70)	1 (25)	175 (69)	0.070
Erector spinae catheter	4 (2)	0 (0)	4 (2)	1.000

ASD, atrial septal defect; MV, mitral valve; PFO, patent foramen ovale; pRBC, packed red blood cells; TV, tricuspid valve.

**Table 3:** Postoperative outcomes.

Parameters	Non-Converted (N = 250)	Converted (N = 4)	Total (N = 254)	*p* Value
**Hospital LOS (day)**	6 (5–8)	6 (6–7)	6 (5–8)	0.195
**Post-operative complications**				
Re-op for bleeding	4 (2)	0 (0)	4 (2)	1.000
Stroke	2 (1)	1 (25)	3 (1)	**0.047**
Renal failure	15 (6)	1 (25)	16 (6)	0.230
Infection	2 (1)	1 (25)	3 (1)	**0.047**
Lymphocele	3 (1)	0 (0)	3 (1)	1.000
**Pre-discharge imaging**				
MR				0.330
None/trace	199 (80)	3 (75)	202 (80)	
Mild	27 (11)	0 (0)	27 (11)	
Moderate	17 (7)	1 (25)	18 (7)	
Severe	0 (0)	0 (0)	0 (0)	
TR				0.957
None/trace	86 (34)	1 (25)	87 (34)	
Mild	97 (39)	2 (50)	99 (40)	
Moderate	54 (22)	1 (25)	55 (22)	
Severe	5 (2)	0 (0)	5 (2)	
**Lymphocele at follow up**	3 (1)	0 (0)	3 (1)	1.000

LOS, length of stay; MR, mitral regurgitation; TR, tricuspid regurgitation.

## Data Availability

The data presented in this study are available on request from the corresponding author. The data are not publicly available due to patient confidentiality.
